# NLRX1 Regulation Following Acute Mitochondrial Injury

**DOI:** 10.3389/fimmu.2019.02431

**Published:** 2019-10-24

**Authors:** Xiaogang Chu, Songwei Wu, Raghavan Raju

**Affiliations:** ^1^Department of Pharmacology and Toxicology, Medical College of Georgia, Augusta University, Augusta, GA, United States; ^2^Department of Anesthesiology and Perioperative Medicine, University of Alabama at Birmingham, Birmingham, AL, United States

**Keywords:** innate immunity, mitochondria, NLRX1, inflammasome, trauma, PAMP, DAMP

## Abstract

Several metabolic, cardiovascular, and neurological disorders are characterized by mitochondrial dysfunction followed by dysregulation of cellular energetics. Mitochondria play an important role in ATP production and cell death regulation. NLRX1, a mitochondria-targeted protein, is known to negatively regulate innate immunity, and cell death responses. However, the role of this protein in cellular homeostasis following mitochondrial injury is not well-understood. To understand the mechanisms underlying the effect of acute injury in regulating NLRX1 signaling pathways, we used an *in vitro* model of mitochondrial injury wherein, rat pulmonary microvascular endothelial cells were subjected to sodium azide treatment or glucose starvation. Both sodium azide and glucose starvation activated NF-κB and TBK1 associated innate immune response. Moreover, increased TBK1, IKK, IκB, and TRAF6 were recruited to mitochondria and interacted with NLRX1. Depletion of endogenous NLRX1 resulted in exacerbated NF-κB and TBK1 associated innate immune response and apoptosis. Our results suggest that NLRX1 participates in the regulation of innate immune response in mitochondria, and plays an important role in the maintenance of cellular homeostasis following acute mitochondrial injury. We propose that the mitochondrial recruitment of inflammatory mediators and their interaction with NLRX1 are protective responses to maintain cellular homeostasis following injury.

## Introduction

Mitochondria are the main source of ATP in the cell. Recent studies suggest that mitochondria is also a master regulator of inflammation (1) and play a key role in injury response (2–4). Mitochondria also participate in a broad range of innate immune pathways, functioning as signaling platforms in cell death and contributing to effector responses (5, 6). Furthermore, they decode incoming danger signals and translate them into appropriate adaptive responses (1). Stressed mitochondria can also be the source of intracellular danger signals that are sensed by the innate immune system to promote sterile inflammation (1). Traumatic injuries cause tissue damage and injured tissues initiate the early host response. The initiating factors and mediators of trauma injuries may use the same innate immune signaling pathways used in immune responses against pathogens (7). Injuries cause cell lysis, macromolecular degradation, and tissue breakdown (necrosis), which release the contents of cells, including damaged mitochondrial DNA (2). During infection, tissue damage, or cell stress, exogenous pathogen-associated molecular patterns (PAMPs) or endogenous “damage”-associated molecular patterns (DAMPs) activate innate immunity through different pattern-recognition receptors (PRRs) including RIG-I-like receptors (RLRs), NOD-like receptors (NLRs), Toll-like receptors (TLR), and C-type lectin receptors (CLRs) (2, 5). PRR ligation activate multiple signaling pathways resulting in the activation of nuclear factor-κB (NF-κB), mitogen-activated protein kinases (MAPKs), and interferon regulatory factors (IRFs), which generate pro-inflammatory cytokines and chemokines for repairing tissue damage and in response to stress (5, 8).

NLRs are a group of intracellular receptors that represent a key component of the host innate immune system (9, 10). The nucleotide-binding domain and leucine-rich repeat–containing protein X1 (NLRX1) is an unusual member of the NLRs family (10). NLRX1 is unique because it represents the first and so far the only example of a PRR family member with a mitochondrial targeting sequence, indicating mitochondrial localization, and providing a fundamental link between mitochondrial function and innate immunity (6, 11, 12). Recent studies suggest that NLRX1 functions as a negative regulator of NF-κB and IRF3 signaling pathway in infection and inflammation (13–15). In addition, some studies implicated NLRX1 in the regulation of cell death, COPD and cancer (16–20). However, the role of NLRX1 in acute cellular injuries remains unknown.

In this manuscript we sought to determine the role of NLRX1 following acute mitochondrial injury and unravel the molecular pathways relating to NLRX1 in the maintenance of cellular homeostasis following injury.

## Results

### Sodium Azide Induced Mitochondrial Injury

**Figure 1:**
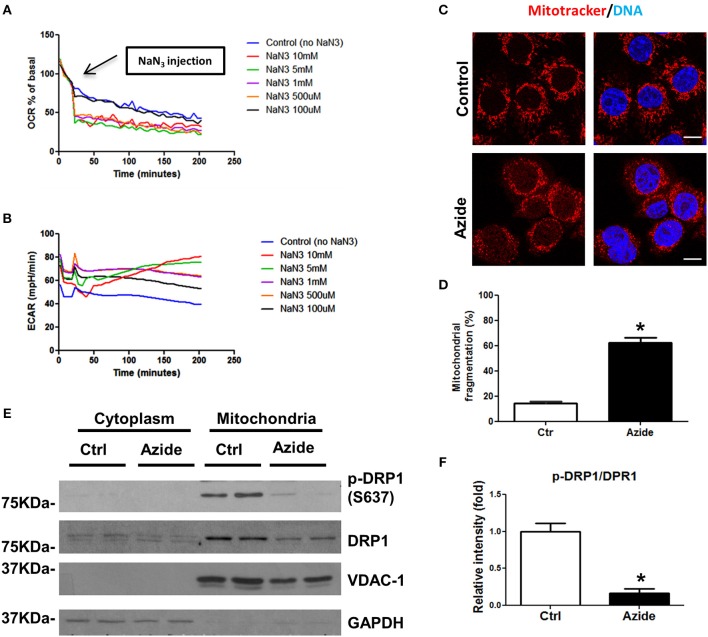
Sodium azide induced mitochondrial injury. **(A)** Representative experiment showing OCR in PMVECs before and after acute injection of various concentrations (0–10 mM) of sodium azide, as measured using Seahorse XFp flux analyzer. Values are expressed as a percentage of the basal rate for each concentration. The data shown is representative of the replicates (*n* = 3). **(B)** Representative experiment showing ECAR in PMVECs as described above. **(C)** Representative confocal images of the mitochondrial morphology in PMVECs. Cells were pre-stained with MitoTracker Red CMXRos and treated with vehicle or sodium azide for 3 h, then fixed with 4% PFA and stained with Hoechst for DNA. Scale bar: 10 μm. **(D)** Quantification of mitochondrial fragmentation of the cells. *n* ≥ 100 cells per experiment. Values represent the mean ± SEM (error bars) from four independent experiments. **(E)** PMVECs were treated with vehicle or 5 mM sodium azide for 3 h, mitochondrial and cytosolic fractions of cells were obtained by using specific lysis buffers and analyzed by western blotting using antibodies against p-DRP1 (S637) and DRP1. VDAC-1 and GAPDH were used as marker of mitochondrial and cytoplasmic fractions. The blots shown are representative of the replicates (*n* = 3). **(F)** Mitochondrial p-DRP1/DRP1 ratio was quantified using Image J software(NIH), * indicates *p* < 0.05 compared to control group.

A number of *in vitro* models have been proposed to mimic injury occurring under pathological conditions (21). Sodium azide, a mitochondrial toxin, can induce a hypoxic-like condition through its ability to inhibit mitochondrial complex IV, which has been widely used to study the cellular mechanisms underlying mitochondria associated damage (22–24). To understand the mechanisms following acute mitochondrial injury, rat pulmonary microvascular endothelial cells (PMVECs) (25, 26) were subjected to azide-induced ATP depletion to model *in vivo* ischemia. Mitochondrial respiration was characterized using the Seahorse extracellular flux (XF) analyzer. In this system mitochondrial oxygen consumption rate (OCR) was used to measure oxidative phosphorylation (OXPHOS) and extracellular acidification rate (ECAR) as a measure of glycolysis. The physiological experiment using Seahorse Analyzer, mitotracker-based cytofluorimetry as well as the protein blot experiments collectively demonstrated the effect of NaN_3−_mediated mitochondrial injury (Figure 1). The dose-response study to test the effect of NaN_3_ on mitochondrial oxygen consumption and ECAR further validated the *in vitro* injury model. OCR was decreased at all concentrations of NaN_3_ tested with the PMVECs, however, an increase in ECAR was observed only at 5 and 10 mM concentrations of NaN_3_, demonstrating efficient blockade of mitochondrial respiration, and active glycolysis at these concentrations (Figures 1A,B). We therefore used 5 mM concentration of NaN_3_ as a model to assess changes in signaling pathways with mitochondrial functional inhibition. Consistent with the decrease in OCR, ATP production was also significantly decreased (data not shown). Next we examined the mitochondrial morphology in PMVECs using fluorescence confocal microscopy. The cells were stained with Mitotracker, a mitochondrial marker. As shown as in Figures 1C,D, mitochondria had tubular or thread-like appearance in control cells. However, in sodium azide treated PMVECs, mitochondrial networks were broken down, and the mitochondria were fragmented into short rods or spheres. To further study the observed phenotypes, we isolated the mitochondrial and cytosolic fractions of control and treated cells and found that the phosphorylation level of Drp1, the mitochondrial dynamics regulation protein, at Ser-637 was decreased with sodium azide treatment (Figures 1E,F), which suggested that sodium azide disrupted the mitochondrial fission-fusion balance and increased fragmentation.

### Sodium Azide or Glucose Starvation-Induced Mitochondrial Injury Activates NF-κB and TBK1 Pathway

**Figure 2:**
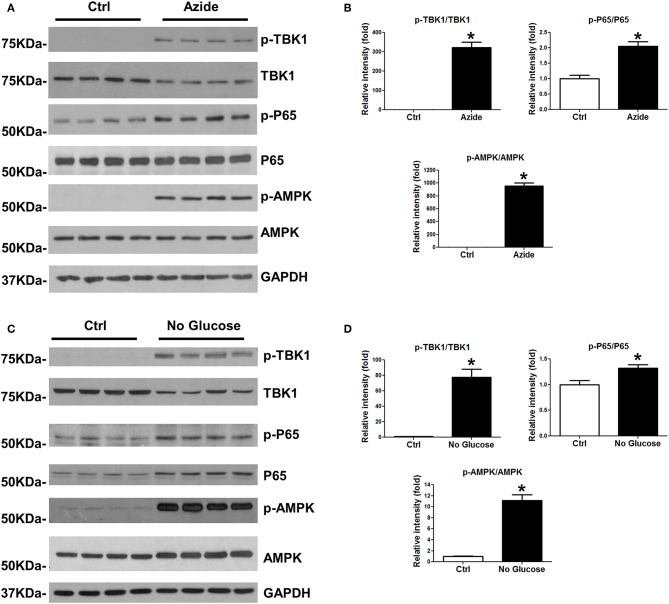
Azide treatment or glucose starvation lead to the activation of TBK1 and NFκB pathways. **(A)** PMVECs were treated with vehicle or sodium azide for 3 h, and subjected to western blot analysis using p-TBK1, TBK1, p-P65, P65, p-AMPK, and AMPK antibodies. GAPDH was used as the loading control. There were 4 samples in each group, the blots shown are representative of the replicates (*n* = 3). **(B)** p-TBK1/TBK1, p-P65/P65, and p-AMPK/AMPK ratios were quantified using Image J software(NIH), * indicates *p* < 0.05 compared to control. **(C)** PMVECs were culture in DMEM with or without glucose overnight; whole cell lysates were subjected to western blot analysis using p-TBK1, TBK1, p-P65, P65, p-AMPK, and AMPK antibodies. GAPDH was used as the loading control. There were 4 samples each group, the blots shown are representative of the replicates (*n* = 3). **(D)** p-TBK1/TBK1, p-P65/P65, and p-AMPK/AMPK ratios were quantified using Image J software(NIH), * indicates *p* < 0.05 compared to control.

Injury-induced immune response is associated with the activation of NF-κB and TBK1/IRF3 signaling pathways. In order to test whether the mitochondrial injury induced by sodium azide-mediated complex IV inhibition could lead to similar results, PMVECs were treated with sodium azide, and assessed phosphorylation and total expression level of TBK1 and P65 proteins; activated TBK1 phosphorylates and activates IRF3 signaling pathway. As shown in Figures 2A,B, p-TBK1, and p-P65 were significantly increased in azide treated cells, which indicated the activation of TBK1 and NF-κB signaling pathways as a result of complex IV inhibition. We also checked the activation of AMPK, one of the central regulators of cellular metabolism in eukaryotes. ATP production decreased by complex IV inhibition elevated AMP/ATP ratio and activated AMPK signaling pathway (Figures 2A,B) (27). We further analyzed the NF-κB, TBK1, and AMPK signaling pathways in glucose deprivation cell culture model to mimic nutrient deprivation observed in ischemic conditions. Apoptosis following azide treatment is well-recognized (23) and our experiments demonstrate that necroptosis also contributes to azide-induced cell death (Supplementary Figure S1).The mitochondrial injury by glucose starvation also led to similar results (Figures 2C,D). Not surprisingly, glucose deprivation also activated increased cell death signaling, as indicated by increased cleaved caspase 3 and cleaved PARP (Supplementary Figure S2).

### TBK1 and IKK Signaling Increased in Mitochondria Following Sodium Azide Induced Mitochondrial Injury

**Figure 3:**
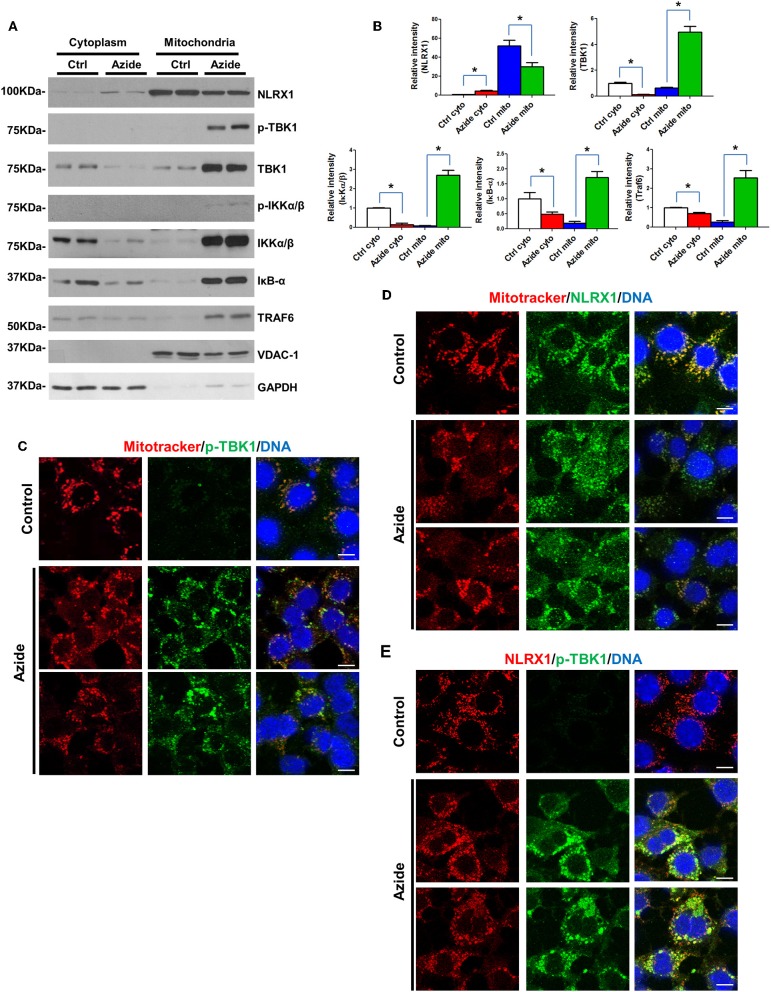
Enhanced TBK1 and IKK signaling in mitochondria following acute mitochondrial injury. **(A)** PMVECs were treated with vehicle or sodium azide for 3 h, mitochondrial and cytosolic fractions of cells were obtained by using specific lysis buffers and analyzed by western blotting using antibodies against NLRX1, p-TBK1, TBK1, p-IKKα/β, IKKα/β, IKB, and TRAF6. VDAC-1 and GAPDH were used as marker of mitochondrial and cytoplasmic fractions. The blots shown are representative of the replicates (*n* = 3). **(B)** Cytosolic and mitochondrial NLRX1,TBK1, IKKα/β, IKB, and TRAF6 intensities were quantified using Image J software(NIH), * indicates *p* < 0.05 compared to control. **(C)** MitoTracker Red CMXRos pre-stained PMVECs were treated with vehicle or sodium azide for 3 h, then fixed with iced methanol and stained with rabbit anti-p-TBK1 and Hoechst. Scale bar: 10 μm. The data shown is representative of the replicates (*n* = 5). **(D)** MitoTracker Red CMXRos pre-stained PMVECs were treated with vehicle or sodium azide for 3 h, then fixed with iced methanol and stained with mouse anti-NLRX1 and Hoechst. Scale bar: 10 μm. The data shown is representative of the replicates (*n* = 5). **(E)** PMVECs were treated with vehicle or sodium azide for 3 h, then fixed with iced methanol and co-stained with mouse anti-NLRX1, rabbit anti-p-TBK1, and Hoechst. Scale bar: 10 μm. The data shown is representative of the replicates (*n* = 5).

Mitochondria are regulatory centers in the innate immune response. A number of studies suggest mitochondria and/or mitochondria-associated membranes (MAMs) play an important role in the initiation of TBK1-IRF3 and IKK-IκB-NF-κB pathways (28–30). To understand the mechanisms underlining the activation of TBK1-IRF3 and IKK-IκB-NF-κB signaling pathways following acute mitochondrial injury, cytoplasmic, and mitochondrial fraction of control and sodium azide treated cells were isolated, and expression level of TBK1-IRF3 and IKK-IκB-NF-κB associated proteins were determined by Western blot. As shown in Figures 3A,B, TBK1, and IKK signaling molecules TBK1, IKKα/β, IκB, and TRAF6 were significantly increased in mitochondria but decreased in cytosol following sodium azide treatment. Interestingly, the phosphorylation level of TBK1 was essentially undetectable in untreated cells, but significantly increased in sodium azide treated cells. Glucose deprivation yielded similar results (Supplementary Figure S3). To confirm these results, we stained PMVECs with a phosphorylated-TBK1-specific antibody; fluorescent immunostaining of control cells showed similar results as WB. In untreated cells p-TBK1 were undetectable and significantly increased in sodium azide treated cells and overlapped with the staining pattern of Mitotracker, the mitochondrial marker (Figure 3C).

We next checked NLRX1, one of the mitochondrial proteins implicated in innate immune response. Consistent with previous reports, NLRX1 was predominantly localized in mitochondria in the control group. However, azide treatment or glucose deprivation resulted in consistent reduction of NLRX1 in mitochondria but not in the cytosol. To confirm these results, we stained PMVECs with an NLRX1-specific antibody, fluorescent immunostaining of control cells showed a punctate pattern of NLRX1 localization that overlapped with the staining pattern of Mitotracker. However, NLRX1 staining was decreased in mitochondria and increased presence was observed in cytoplasm when compared to untreated cells (Figures 3A,D).

We also co-stained p-TBK1 and NLRX1 in PMVECs, NLRX1-p-TBK1 co-localization is shown in Figure 3E. These data suggest that acute mitochondrial injury by sodium azide or glucose starvation led to significant activation of TBK1 and IKK signaling in mitochondria. And the co-localization of p-TBK1 and NLRX1 suggested NLRX1 may play an important role in TBK1 signaling pathway in mitochondria.

### Interaction of NLRX1, TBK1, and IKK in Mitochondrial Injury Induced by Complex IV Inhibition

**Figure 4:**
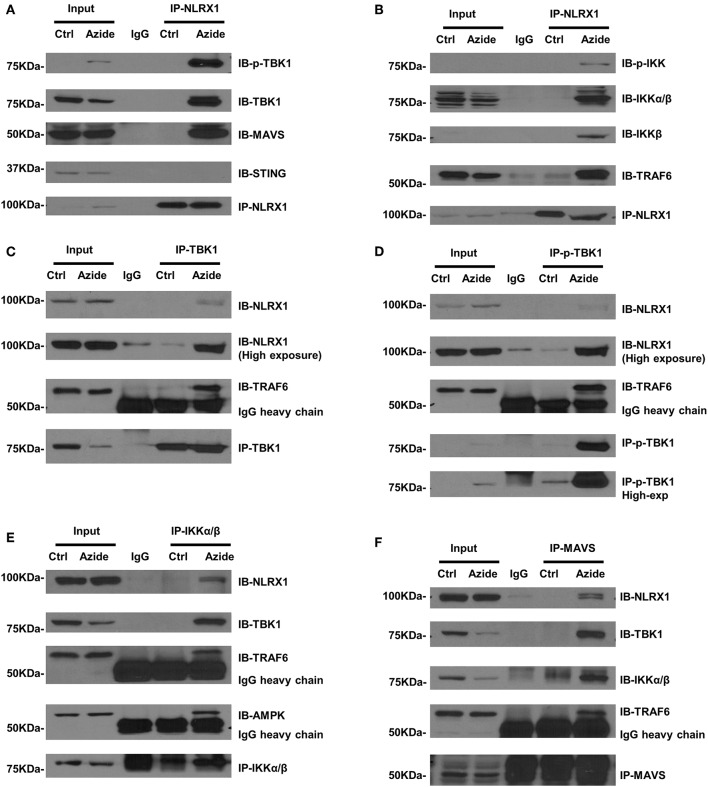
Association of NLRX1 with TBK1 and IKK pathways. **(A)** Co-immunoprecipitation (co-IP) of endogenous NLRX1 with p-TBK1, TBK1, MAVS, and STING. PMVECs were treated with vehicle or sodium azide for 3 h and Cell lysates were subjected to immunoprecipitation using mouse anti-NLRX1 antibody or mouse IgG. The immunoprecipitates were separated by SDS-PAGE and blotted with p-TBK1, TBK1, MAVS, STING, and NLRX1 antibodies. The blots shown are representative of the replicates (*n* = 4). **(B)** Co-IP of endogenous NLRX1 with p-IKK, IKKα/β, IKKβ, and TRAF6. Cell treatment, co-IP and Western blot (WB) were performed as described above. The blots shown are representative of the replicates (*n* = 3). **(C)** Co-IP of endogenous TBK1 with NLRX1 and TRAF6. PMVECs were treated with vehicle or sodium azide for 3 h and Cell lysates were subjected to immunoprecipitation using rabbit-anti-TBK1 antibody or rabbit IgG. The immunoprecipitates were separated by SDS-PAGE and blotted with NLRX1, TRAF6, and TBK1 antibodies. The blots shown are representative of the replicates (*n* = 3). **(D)** Co-IP of endogenous p-TBK1 with NLRX1 and TRAF6. Cell treatment, co-IP and Western blot (WB) were performed as described above. The blots shown are representative of the replicates (*n* = 2). **(E)** Co-IP of endogenous IKKα/β with NLRX1, TRAF6, and AMPK. Cell treatment, co-IP and Western blot (WB) were performed as described above. The blots shown are representative of the replicates (*n* = 3). **(F)** Co-IP of endogenous MAVS with NLRX1, TBK1, IKKα/β, and TRAF6. Cell treatment, co-IP and Western blot (WB) were performed as described above. The blots shown are representative of the replicates (*n* = 3).

Although NLRX1 has been suggested to be a negative regulator of NF-κB and TBK1 signaling pathway, the molecular mechanisms remain unclear. Given the enhanced TBK1 and IKK signaling in mitochondria and the co-localization of NLRX1 and p-TBK1 as established above, we sought to examine the protein-protein interactions by endogenous co-immunoprecipitation. To test whether NLRX1 can interact with endogenous TBK1, control or azide treated PMVEC lysates were immunoprecipitated with an isotype IgG or anti-NLRX1 antibody, followed by western blot analysis with anti-TBK1, anti-p-TBK1, anti-MAVS, and STING antibodies. The specificity of immunoprecipitation was demonstrated by the lack of interaction of NLRX1 with a mitochondrial membrane protein VDAC1 (Supplementary Figure S4). As shown in Figure 4A, in control cells, STING could not be co-immunoprecipitated with NLRX1, however, there was detectable levels of TBK1, p-TBK1, and MAVS following complex IV inhibition. To test whether NLRX1 can interact with IKK and TRAF6, control, or azide treated PMVECs lysates were immunoprecipitated with an IgG or anti-NLRX1 antibody, followed by western blot analysis with the p-IKK, IKKα/β, IKKβ, and TRAF6 antibodies. p-IKK, IKKα/β, IKKβ, and TRAF6 could be co-immunoprecipitated with NLRX1 in sodium azide treated cells but not in untreated cells (Figure 4B). To confirm the specificity of the interaction between NLRX1 and TBK1, we co-immunoprecipitated the cell lysates with TBK1 or p-TBK1 antibodies, followed by western blot analysis with NLRX1 and found that NLRX1 was associated with both TBK1 and p-TBK1 (Figures 4C,D). To further verify the specificity of the interaction between NLRX1 and IKK, we co-immunoprecipitated the cell lysates with IKK antibody, followed by western blot analysis with the NLRX1 (Figure 4E). To verify the specificity of the interaction between NLRX1 and MAVS, we co-immunoprecipitated the cell lysates with MAVS antibody, followed by western blot analysis with NLRX1, TBK1, IKKα/β, and TRAF6 and found that NLRX1, TBK1, IKKα/β, TRAF6, and NEMO could be co-immunoprecipitated with MAVS in sodium azide treated cells (Figure 4F, Supplementary Figure S5). To further confirm the association of NLRX1 and TBK1 or IKK signaling molecules, we performed immunoprecipitation analysis in LPS stimulated RAW 264.7 cells. We could reproducibly detect a portion of TBK1 or IKK signaling molecules in the NLRX1 immunoprecipitates (Supplementary Figure S6).

### NLRX1 Attenuates Inflammation and Cell Death

**Figure 5:**
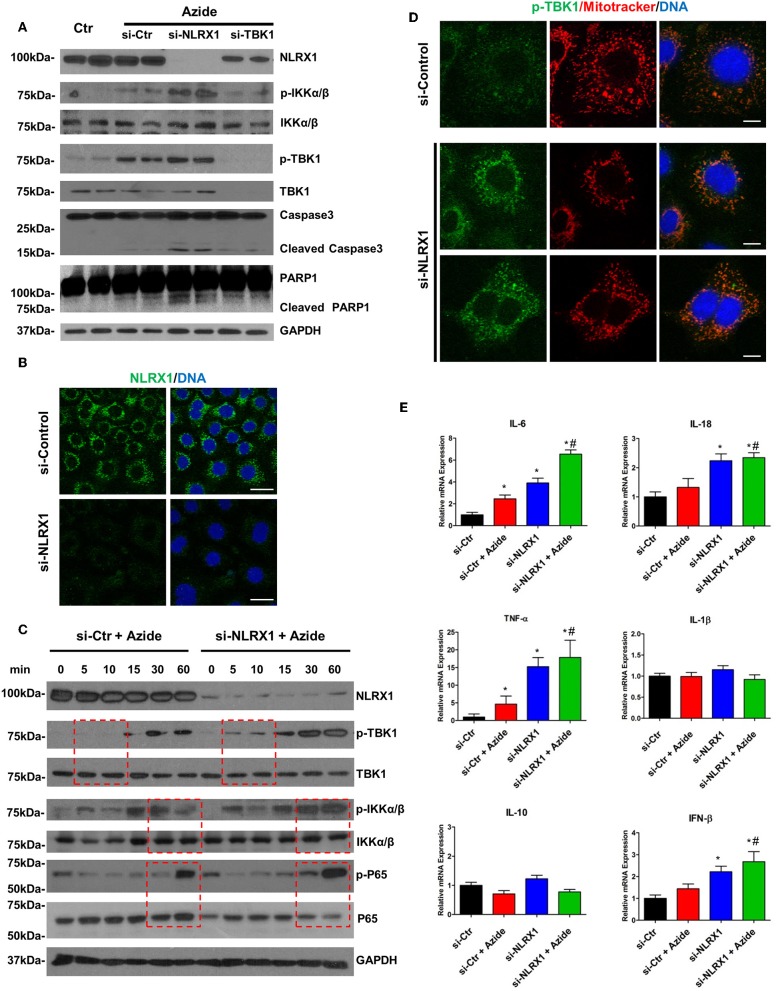
Depletion of endogenous NLRX1 lead to increase of apoptosis and innate immune response. **(A)** PMVECs were transfected with 50 nM NLRX1, TBK1, or control siRNA for 72 h, and treated with vehicle or sodium azide for 2 h before cell lysis. Cell lysates were subjected to SDS-PAGE and immunoblotting using NLRX1, p-IKK, IKK, p-TBK1, TBK1, caspase 3, and PARP1 antibodies. GAPDH was used as the loading control. There were two samples each group, the blots shown are representative of the replicates (*n* = 4). **(B)** PMVECs were transfected with 50 nM NLRX1 or control siRNA, 72 h after the transfection; cells were fixed with iced methanol and stained for NLRX1 and DNA. Scale bar: 20 μm. The data shown is representative of the replicates (*n* = 5). **(C)** PMVECs were transfected with 50 nM NLRX1 or control siRNA for 72 h, and treated with sodium azide for indicated period of time before lysis. Cell lysates were subjected to SDS-PAGE and immunoblotting using NLRX1, p-IKK, IKK, p-TBK1, TBK1, p-P65, and P65 antibodies. GAPDH was used as the loading control. The blots shown are representative of the replicates (*n* = 3). **(D)** PMVECs were transfected with 50 nM NLRX1 or control siRNA for 72 h, and pre-stained with MitoTracker Red CMXRos for 30 min. Cells were fixed with iced methanol and stained for p-TBK1 and DNA. Scale bar: 10 μm. The data shown is representative of the replicates (*n* = 5). **(E)** SYBR green real-time PCR amplification of IL-6, IL-10, IL-1β, TNF-α, IL-18, and IFN-β. PMVECs were transfected with 50 nM NLRX1 or control siRNA for 72 h, and treated with vehicle or sodium azide for 3 h. * indicates *p* < 0.05 compared to control siRNA group, # indicates *p* < 0.05 compared to azide treated control siRNA group.

Both NF-κB and TBK1 have been suggested to be regulatory molecules in apoptotic cell death and inflammation (31, 32). The interaction of NLRX1 with activated IKK or TBK1 may be a protective response under cell stress. To assess the function of NLRX1 in complex IV inhibition-induced mitochondrial injury, specific siRNA was used to effectively deplete endogenous NLRX1. The diminution of NLRX1 signal on immunoblots and staining in cells verifies efficient NLRX1 knockdown (Figures 5A,B). Compared with control, WT cells treated with sodium azide showed increased level of p-IKKα/β and p-TBK1. In NLRX1 knocked down cells there was further increase in the level of both p-IKKα/β and p-TBK1 demonstrating an inflammatory phenotype with NLRX1 deficiency (Figure 5A). In cells in which NLRX1 expression had been suppressed, the expression level of cleaved caspase 3 and cleaved PARP were also significantly increased indicating that NLRX1 deficiency not only exacerbate inflammation but also enhance apoptosis (Figure 5A). Interestingly, we observed decreased NLRX1 protein level in TBK1 depleted cells (Figure 5A). Mitochondrial respiration study using Seahorse did not demonstrate any significant change in OCR following NLRX1 deficiency (Supplementary Figure S7).

To evaluate the effect of NLRX1 on NF-κB signaling pathway, PMVECs were treated with sodium azide for up to 1 h and the amounts of total and phosphorylated IKKα/β and P65 were assessed. Sodium azide exposure resulted in the increase of both p-P65 and p-IKKα/β in the WT cells. This process was accelerated or enhanced in NLRX1 depleted cells. A higher amount of p-P65 was detected in NLRX1 depleted PMVEC by 30 min of sodium azide exposure (Figure 5C). In the case of p-IKKα/β, the levels were higher at 30 and 60 min in NLRX1 depleted cells. We also evaluated the effect of NLRX1 depletion on TBK1 signaling pathway. Similar to the findings in NF-κB, after sodium azide exposure, p-TBK1 was markedly increased in the WT cells. This process was accelerated in NLRX1 depleted cells, a higher amount of p-TBK1 was detected in NLRX1 depleted PMVECs within 5–10 min of sodium azide stimulation. These results suggest that in addition to serving as an attenuator of NF-κB, NLRX1 also inhibits sodium azide-induced activation of IFN-I responses. To confirm the inhibition effect of NLRX1 on TBK1, we examined p-TBK1 in PMVECs using immunofluorescence confocal microscopy. As shown in Figure 5D, compared with control siRNA treated cells, p-TBK1 was significantly increased and co-localized with mitotracker in mitochondria following NLRX1 siRNA treatment.

To further confirm the role of NLRX1 in NF-κB and TBK1 signaling pathway NF-κB and TBK1 regulated cytokines were evaluated in WT and NLRX1 knockdown cells using real-time PCR. Consistent with the western blot results, mitochondrial injury resulted in an increase in pro-inflammatory cytokines IL6, IL18, TNFα, and IFNβ mRNA in both WT and NLRX1 KD cells. Compared with WT cells, NLRX1 KD cells had significantly higher expression level IL6, IL18, TNFα, and IFNβ in both sodium azide treated and untreated cells (Figure 5E).

## Discussion

The dysregulation of cellular energetics due to mitochondrial injury is a hallmark of metabolic and cardiovascular diseases. Though development of an *in vitro* model that exactly mimic mitochondrial dysfunction in human diseases is challenging, a highly reductionistic model wherein the respiratory complex IV is inhibited by sodium azide was employed by several investigators (23). We used this model to test the inter-relationship between mitochondrial injury, NLRX1 and inflammation. Hypoxia as well as chemical inhibition of oxidative phosphorylation (OXPHOS) switch cells from mitochondrial oxidation to glycolysis for ATP production. This was evident when PMVECs were treated with sodium azide. Though a decrease in oxygen consumption was observed at lower azide concentrations, at 5 and 10 mM concentrations there was an increase in glycolytic rate as shown by elevated ECAR demonstrating significant decline in OXPHOS. Furthermore, the decline in mitochondrial function induced by complex IV inhibition was followed by a decrease in the level of mitochondrial NLRX1.

The increased mitochondrial fission observed following azide treatment can be attributed to OXPHOS inhibition. Nevertheless, the mitochondrial injury due to complex IV inhibition triggered inflammatory and innate immune signaling response as evident from the increased p-TBK1 and p-P65. A similar effect was observed on glucose starvation, which is not surprising as it is known that nutrient deprivation can activate ER stress and inflammatory response (33, 34).

Mitochondria associated protein NLRX1 has been suggested to be a negative regulator of NF-κB and IRF3 signaling pathway, though the molecular mechanisms remain unclear. However, TBK and IKK pathway signaling molecules TBK1, IKKβ, IKB, and Traf6 were localized predominantly in the cytoplasm. The influx of signaling molecules participating in TBK and IKK pathway into mitochondria, as well as an alteration in the level of NLRX1 following sodium azide treatment suggest that mitochondria play an important role in the control of inflammation. Consistent with our results, there have been previous reports of mitochondrial localization of p-TBK1, IKKα/β, and IκB (30, 35, 36). The intramitochondrial localization of NLRX1 remains controversial (11, 37, 38). Nevertheless, in conditions of mitochondrial injury such as that in ischemic or chemical insult, the trafficking dynamics are likely to be altered and NLRX1 localization within mitochondria cannot be predicted to be the same as in normal conditions. Though further investigations are required to identify the sub-mitochondrial localization, the molecular mechanism of recruitment of these molecules and protein trafficking dynamics in response to mitochondrial injury, the observation that a set of complex inflammatory mediators are increased in the mitochondria following injury is novel. Interestingly this is observed not only following complex IV inhibition, but also following glucose deprivation. The mitochondrial recruitment of these mediators are likely to play a critical role in the mitochondrial control of inflammation. This is important toward understanding the inflammatory response observed following hypoxic or pseudohypoxic situation observed following injury or aging and the role of mitochondria in this adaptive response (39).

The azide treatment resulted in the reduction of NLRX1 in mitochondria. Whereas, the moderate decrease in NLRX1 in the mitochondria following sodium azide treatment was associated with a proinflammatory response; a more complete elimination of NLRX1 by siRNA treatment demonstrated a robust inflammatory response substantiating the reports that NLRX1 is a negative regulator of inflammation. This is consistent with the finding that LPS stimulation of macrophages deficient in NLRX1 led to increased amount of IL-6 and IFN-β compared to similarly stimulated wild type cells (13). We also found that following complex IV inhibition, NLRX1 failed to interact with STING whereas NLRX1 could be immunoprecipitated with TBK1. According to a previous observation NLRX1 interaction with STING is necessary to disrupt TBK1-STING signaling and reduce innate response to HIV-1 and DNA viruses (15). In our model, it is likely that NLRX1 is interacting with TBK1 directly or through another protein participating in this complex, however the mechanism by which this interaction modulates TBK1 needs to be further examined. Nevertheless, in mitochondria we localized many of the molecular participants in the NLRX1-dependent inflammatory pathway, including p-IKK, p-TBK1, TRAF-6, and MAVS, and with the exception of STING, all of them were co-immunoprecipitated with NLRX1.

Our studies further demonstrated that in the absence of NLRX1, complex IV inhibition led to increased apoptotic process, as indicated by increased cleaved caspase 3 and cleaved PARP. A temporal increase in p-TBK1 was observed in the absence of NLRX1, along with increased level of NFκb subunit p-p65. Consistent with this, gene expression of a number of proinflammatory cytokines were also elevated with complex 1V inhibition in NLRX1 knockdown cells. Interestingly IL-10 expression did not show any significant change. We also observed that in the absence of TBK1, the protein level of NLRX1 decreased, and the mechanism needs to be further examined.

Though the cause-effect relationship between inflammation and tissue injury is unclear, recent reports suggested a role for host-derived DAMPs in the inflammatory response (40, 41). Moreover, DAMPS of mitochondrial origin as an inflammatory mediator is well-reported (2, 42). Pattern recognition receptors such as RLH and NLR family of proteins are important in the recognition of PAMPs and DAMPS and the trigger of innate immune response. NLRX1 has been reported to attenuate the inflammatory response by negatively regulating RIG1-MAVS interaction. However, MAVS-dependent anti-viral signaling has been demonstrated to be cell-specific and strain (phenotypic difference in NLRX1 KO mice generated by different groups) specific as demonstrated by several groups of investigators (13, 43, 44). Furthermore, the ability of NLRX1 to associate with MAVS, which is an outermembrane protein, depends on its own localization (matrix vs. outer membrane) as reported by other laboratories (13, 37). More recent reports suggest that NLRX1 also interacts with critical mediators involved in both TBK1 and IKK mediated inflammatory response (14, 15). Though the role of NLRX1 in the regulation of antiviral signaling was reported by many laboratories, its role in innate response triggered by tissue injuries remains unclear. Nevertheless, mitochondrial functional decline is a common denominator in many of models of injury and ischemia. Furthermore, irreversible tissue damage occurs when supplies of ATP are not sufficient to maintain cellular function (45). Sodium azide is known to inhibit oxidative phosphorylation via inhibition of complex IV, a critical enzyme in the mitochondrial electron transport chain, thereby resulting in a rapid depletion of intracellular ATP (46, 47). Using this model we show that, following mitochondrial injury, p-IKK, p-TBK1, TRAF-6, and MAVS are recruited to mitochondria and interact with NLRX1. We predict that the mitochondrial recruitment of inflammatory mediators and their interaction with NLRX1 are adaptive protective responses following injury in order to sequester NLRX1 from inhibiting the injury-induced inflammation.

## Experimental Procedures

### Cell Culture and Drug Treatment

Rat pulmonary microvascular endothelial cells (PMVECs) were a kind gift from the University of South Alabama. PMVECs were maintained as subconfluent monolayers in Dulbecco's Modified Eagle's medium (Gibco) supplemented with 10% fetal bovine serum (HyClone) and 100 units/ml penicillin plus 100 μg/ml streptomycin (Invitrogen) at 37°C in 5% CO_2_. For drug treatment experiments, cells were treated with sodium azide in DMEM in the presence or absence of glucose (Gibco) for the durations indicated.

### Immunoblotting Analysis

Immunoblotting procedures were performed as we described previously (48). Briefly, cells were homogenized in the lysis buffer. Lysates were clarified at 12,000 g for 10 min at 4°C, and protein concentrations were determined by the Bradford protein assay (Bio-Rad Laboratories, Hercules, CA). Equal amounts of protein were loaded onto 8–12% SDS–PAGE, transferred onto polyvinylidene difluoride membranes, probed with the indicated primary antibody and the appropriate secondary antibody conjugated with horseradish peroxidase (Cell Signaling), and the immune complexes were detected by standard methods. The antibodies used in this study are as follows: Mouse monoclonal NLRX1 (EMD Millipore, 1:3,000), p-DRP1(S637) (Cell Signaling,1:1,000), Caspase3 (Cell Signaling,1:1,000), GAPDH (Cell Signaling,1:3,000), p-P65 (Cell Signaling,1:1,000), P65 (Cell Signaling,1:1,000), p-IκB-α (Cell Signaling 1:500), IκB-α (Cell Signaling,1:1,000), p-TBK1 (Cell Signaling,1:1,000), TBK1 (Cell Signaling,1:1,000), p-AMPK (Cell Signaling,1:1,000), AMPK (Cell Signaling,1:1,000), STING (Cell Signaling,1:1,000), IKKβ (Cell Signaling,1:1,000), p-IKK α/β (Cell Signaling,1:300), DRP1 (Santa Cruz,1:1,000), IKKα/β (Santa Cruz,1:500), TRAF6 (Santa Cruz,1:1,000), MAVS (Santa Cruz,1:1,000), PARP1 (Santa Cruz,1:1,000), β-Actin (Abcam,1:3,000), VDAC1 (Abcam,1:1,000), RIP1 (Cell signaling, 1:1,000), and RIP3 (Santa Cruze, 1:1,000). HRP linked anti-rabbit or anti–mouse IgG second antibodies (Cell Signaling,1:10,000).

### Mitochondrial and Cytoplasmic Isolation

Mitochondria were isolated using the mitochondria isolation kits for cultured cells (AbCam, Cambridge, UK) and (BioVision, Milpitas, CA), according to the manufacturer's instructions.

### Mitochondrial Respiration Assay

Mitochondrial respiration was performed as we described previously (49). Briefly, PMVECs were plated at 1.5 × 10^4^ cell/well overnight in specialized XFp miniplate. The following day, growth medium was replaced with minimal DMEM supplemented with 10.0 mM glucose, 1.0 mM sodium pyruvate, and 2.0 mM L-glutamine, pH adjusted to 7.4 with NaOH. The plate was incubated at 37°C for 30 min without CO2 before beginning the assay. After the mini plate was loaded, the Seahorse XFp analyzer performed a routine equilibration of 12 min, followed by 4 basal measurements, an acute azide injection (0–10 mM), and then 33 additional measurements at 5 min intervals. For each concentration, post injection values have been normalized and expressed as a percentage of the average of the first four basal values.

### Real-Time Polymerase Chain Reaction

Total RNA was isolated using TRIZOL reagent (Thermo Fisher Scientific, Waltham, MA) and cDNA was synthesized using ImProm-II™ Reverse Transcription System (Promega, WI). Realtime PCR was performed as described previously (50). The sequences of the primers used were: IL-1β: Forward: CCCTGCAGCTGGAGAGTGTGG, Reverse: TGTGCTCTGCTTGAGAGGTGCT, IL-18: Forward: CAGACCACTTTGGCAGACTTCACT, Reverse: GGATTCGTTGGCTGTTCGGTCG, IFN-β: Forward: CACGCCGCGTCTTGGT, Reverse: TCTAGGCTTTCAATGAGTGTGCC, IL-6: Forward: GAGCCCACCAGGAACGAAA, Reverse: AACTGGCTGGAAGTCTCTTGC; IL-10: Forward: TGCGACGCTGTCATCGATTT, Reverse: GTAGATGCCGGGTGGTTCAA; TNF-α: Forward: ACGTCGTAGCAAACCACCAA; Reverse: GCAGCCTTGTCCCTTGAAGA; β-actin: Forward: AGTACCCCATTGAACACG; Reverse: AATGCCAGTGGTACGACC. The results are expressed after normalizing to the values obtained for samples in control group.

### Small Interfering RNA (siRNA)-mediated Knockdown

For the siRNA studies, the 21-mers of the siRNA duplexes against NLRX1, TBK1, and control were synthesized by Dharmacon Research Inc. (Lafayette, CO). The two single-stranded RNAs (20 μM) were annealed to one another by incubation in annealing buffer for 1 min at 90°C, followed by 1 h at 37°C. Transfections of PMVECs were carried out with siRNA in a 24-well plate using Lipofectamine RNAiMAX (Invitrogen), according to the manufacturer's recommendations. The siRNAs used were:

ON-TARGETplus RAT NLRX1 (L-101379-02-005)
Target 1: GCAGGAAACACUUCGGUGA
Target 2: CUGCCCAGCUGGACCGUAA
Target 3: CAAUUUAGCAGGUGUGCGA
Target 4: CCAAAGGCAUCGUCGGAAC
ON-TARGETplus RAT TBK1 (L-101406-02-005)
Target 1: GGGAACAUCAUGCGCGUCA
Target 2: CUAGAGAGUUAGAGGACGA
Target 3: AUCAAGAACUAAUGCGGAA
Target 4: UCACAGAGAUUUACUAUCA.

### Indirect Immunofluorescence and Imaging

PMVECs were grown on glass coverslips and fixed using cold methanol or 4% PFA. Fixed cells were blocked with 1% BSA/10% normal goat serum in PBS for 1 h, and then incubated in 4°C overnight with the primary antibodies (mouse anti-NLRX1 1:300; rabbit anti-p-TBK1 1:100). Cells were then washed and incubated for 1 h with the DNA stain Hoechst and goat anti-rabbit or anti-mouse secondary antibodies coupled to Alexa 488 or Alexa 594 (Invitrogen) (1:1,000). A SlowFade AntiFade kit (Invitrogen) was used to inhibit photobleaching. Cells were imaged using an oil objective on a Zeiss 780 Upright Confocal (Carl Zeiss). Images were collected and processed with Adobe Photoshop for publication.

### Mitochondria Staining

Cells were incubated with 100 nM MitoTracker®Red CMXRos (Cell Signaling) under normal culture conditions for 30 min, then washed with PBS and incubated under normal culture conditions for 60 min. After incubation, cells were fixed and then rinsed 3 times with PBS for 5 min. Mitochondria were subsequently visualized by fluorescence confocal microscopy using a Zeiss 780 Upright Confocal (Carl Zeiss).

### Co-immunoprecipitation

Co-Immunoprecipitation was performed as described previously (51). In brief, PMVECs were lysed in IP lysis buffer (Pierce, Rockfort, IL) and equal amounts of cell lysate were incubated with 2 μg primary antibody at 4°C overnight, Rec-Protein G-Sepharose (Invitrogen, Fredrick, MD) or Pierce Protein A/G plus Agarose (Thermo Scientific™), blocked with 5% BSA (Sigma-Aldrich), were added, and the mixture was incubated for 60 min at 4°C. Immunoprecipitates were washed four times with IP wash buffer and separated by SDS-PAGE and transferred onto membrane to perform western blotting.

### Statistics

Data are presented as mean ± S.E.M. for replicates as indicated. Student *t*-test or One-way ANOVA, were used for statistical analysis using Prism 6 (GraphPad Software). *P* < 0.05 was considered to be statistically significant.

## Data Availability Statement

All datasets generated for this study are included in the manuscript/Supplementary Files.

## Author's Note

The content is solely the responsibility of the authors and does not necessarily represent the official views of the National Institutes of Health.

## Author Contributions

XC designed, performed, and interpreted most of the experiments and participated in writing of the manuscript. SW participated in the discussion and editing of the manuscript as well as in the use of the PMVECs. RR took part in conceptualization, planning, interpretation of the data, and also wrote, edited and finalized the manuscript.

### Conflict of Interest

The authors declare that the research was conducted in the absence of any commercial or financial relationships that could be construed as a potential conflict of interest.

## Abbreviations

NLRX1: Nucleotide-binding domain and leucine-rich repeat–containing protein X1
DAMPs: Damage associated molecular patterns
PAMPs: Pathogen associated molecular patterns
PRR: Pattern recognition receptor
CLR: C-type lectin receptors
PMVEC: pulmonary microvascular endothelial cell.
