# Supplementary material for: NLRX1 Regulation Following Acute Mitochondrial Injury
Source: Front Immunol. 2019 Oct 24;10:2431. doi: 10.3389/fimmu.2019.02431 (PMC6830126; doi:10.3389/fimmu.2019.02431)
Supplement: Supplementary file 1 [file Presentation_1.pdf]

Supplementary figure S1
Necroptosis following acute mitochondrial injury

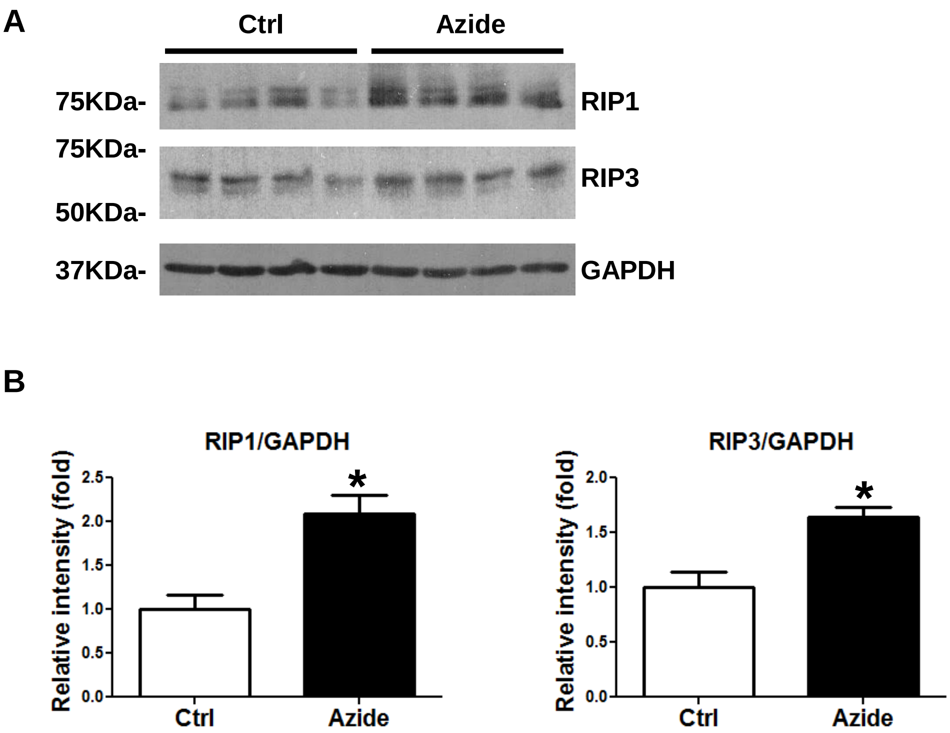

Supplementary figure S2
Increased apoptosis following glucose starvation

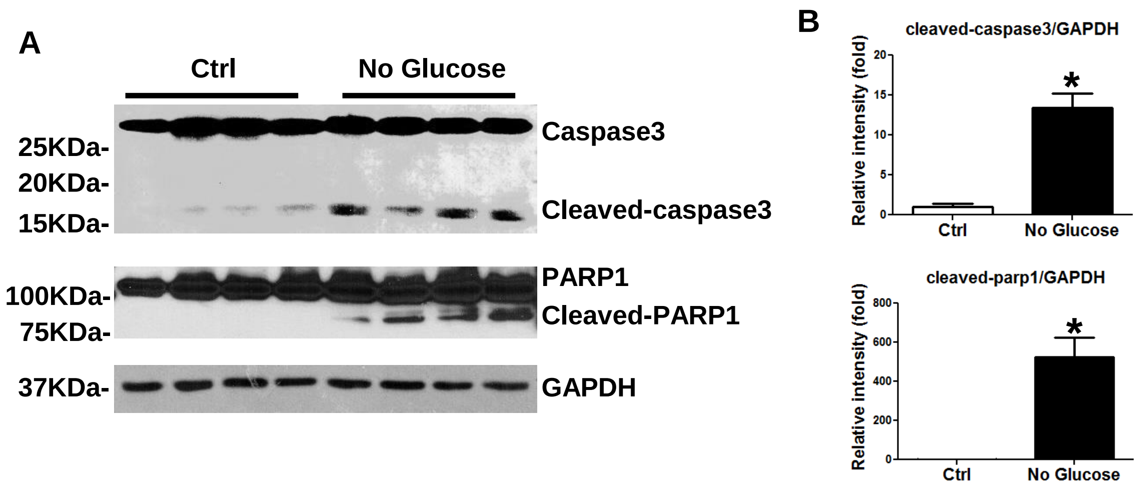

Supplementary figure S3
Increase of TBK1 and Traf6 in mitochondria following glucose starvation

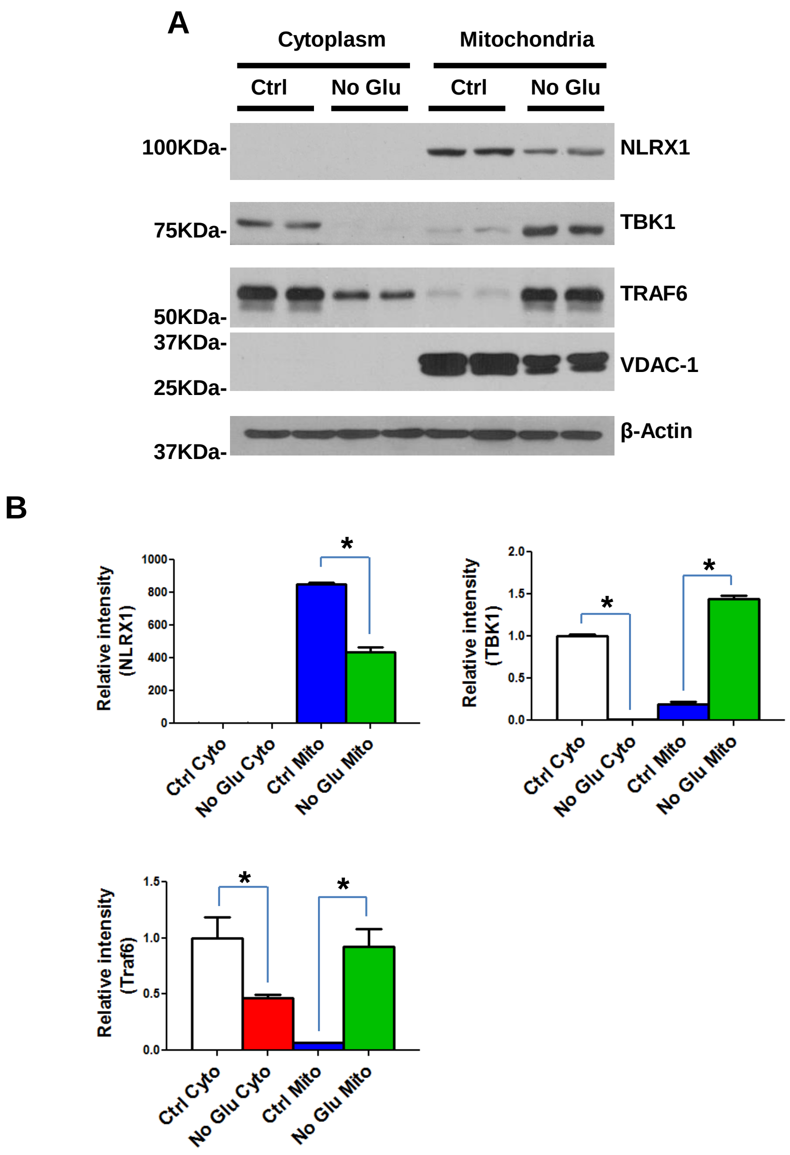

Supplementary figure S4
Co-immunoprecipitation (co-IP) of endogenous NLRX1 with VDAC1

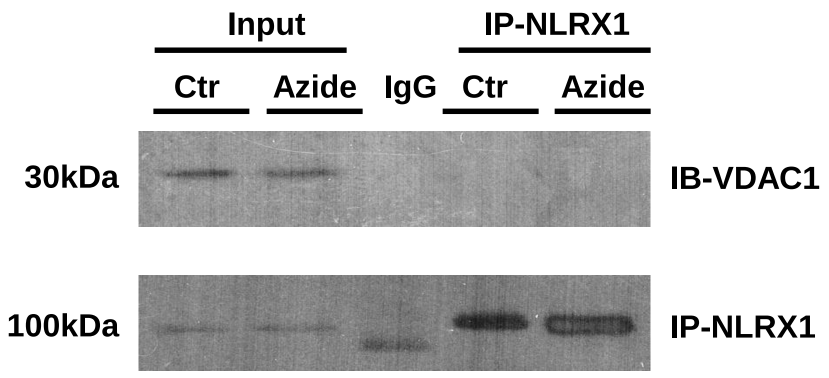

Supplementary figure S5
Association of NLRX1 with NEMO

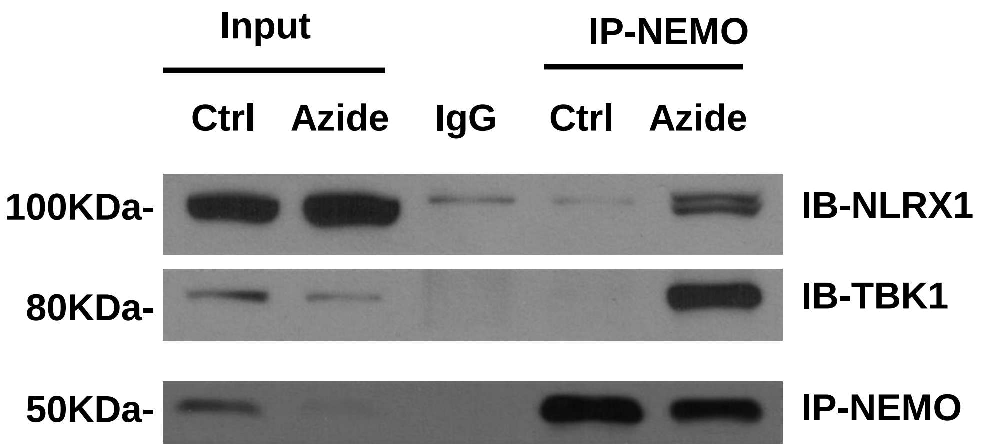

Supplementary figure S6
Association of NLRX1 with TBK1 and IKK pathways following LPS treatment

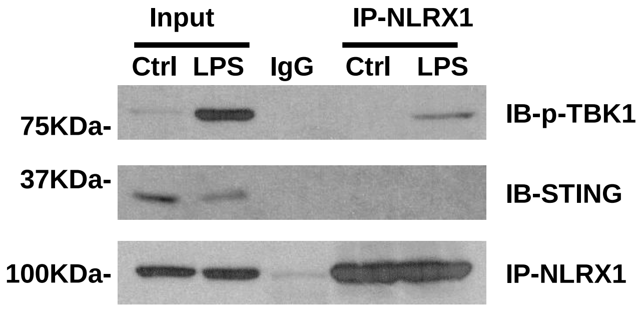

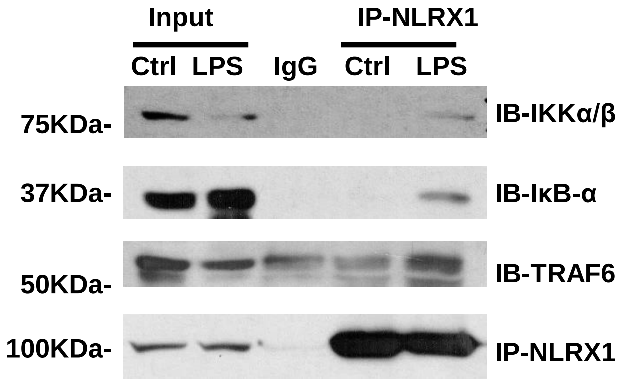

Supplementary figure S7
Mitochondrial respiration following NLRX1 KD

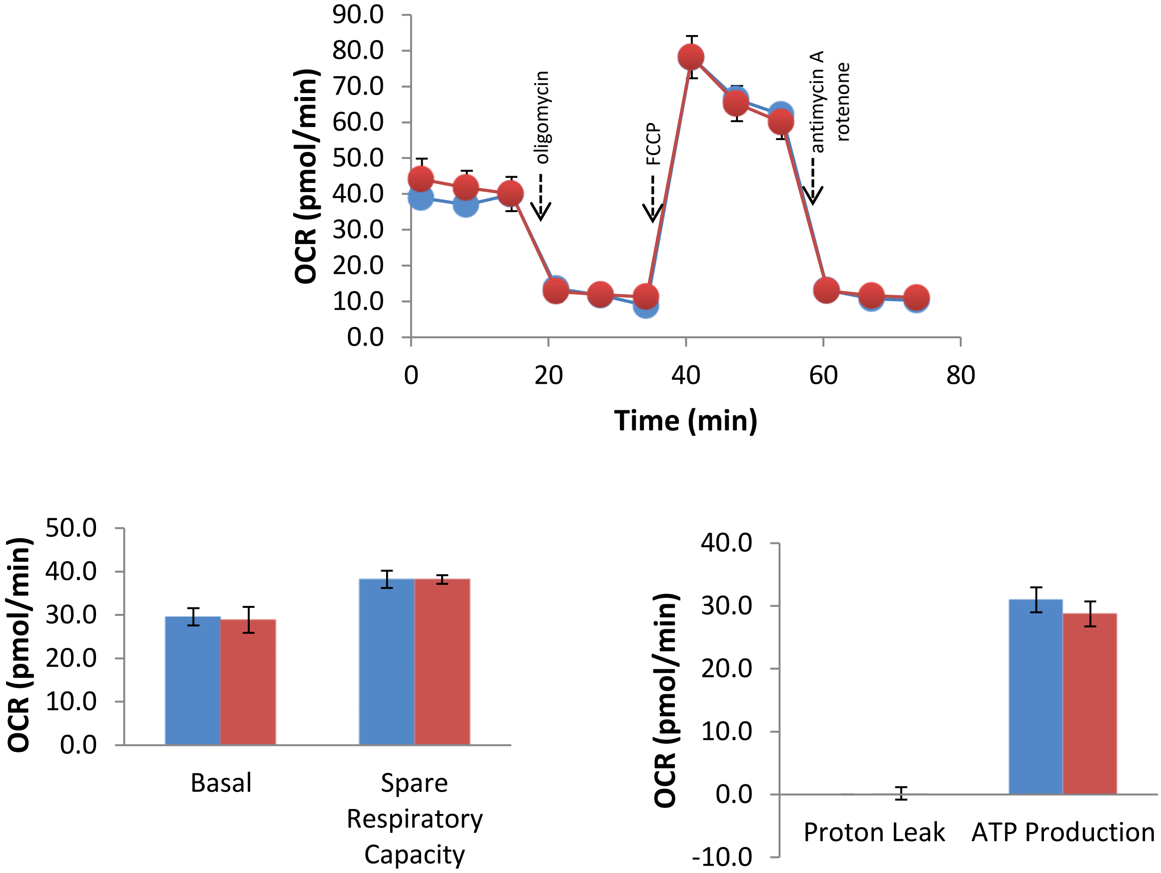

Supplementary figure S1. Necroptosis following acute mitochondrial injury

A. PMVECs were treated with vehicle or sodium azide for 3 hours, and subjected to western blot analysis using RIP1 and RIP3 antibodies. GAPDH was used as the loading control.
B. RIP1/GAPDH and RIP3/GAPDH ratios were quantified using Image J software (NIH), * indicates $p<0.05$ compared to control.

Supplementary figure S2. Increased apoptosis following glucose starvation

A. PMVECs were cultured in DMEM with or without glucose overnight, and subjected to western blot analysis using caspase 3and PARP1 antibodies. GAPDH was used as the loading control. There were 4 samples each group, the blots represent best of the replicates (n=2).
B. Cleaved-caspase3/GAPDH and cleaved-PARP1/GAPDH ratios were quantified using Image J software (NIH), * indicates $p<0.05$ compared to control.

Supplementary figure S3. Increase of TBK1 and Traf6 signaling in mitochondria following glucose starvation

A. PMVECs were cultured in DMEM with or without glucose overnight; mitochondrial and cytosolic fractions of cells were obtained by using specific lysis buffers and analyzed by western blotting using antibodies against NLRX1, TBK1 and TRAF6. VDAC-1 and β-actin was used as the loading control. The blots represent best of the replicates (n=2).
B. Cytosolic and mitochondrial NLRX1, TBK1 and TRAF6 intensities were quantified using Image J software (NIH), * indicates $p<0.05$ compared to control.

Supplementary figure S4. Co-immunoprecipitation (co-IP) of endogenous NLRX1 with VDAC1

PMVECs were treated with vehicle or sodium azide for 3 hours and Cell lysates were subjected to immunoprecipitation using mouse anti-NLRX1 antibody or mouse IgG. The immunoprecipitates were separated by SDS-PAGE and blotted with VDAC1 and NLRX1 antibodies.

Supplementary figure S5. Association of NLRX1 with NEMO

PMVECs treated with azide or vehicle for 3 hours then lysis with IP buffer; 800ug cell lysis co-IP with NEMO antibody or mouse IgG. The immunoprecipitates were separated by SDS-PAGE and blotted with NEMO, TBK1 and NLRX1 antibodies.

Supplementary figure S6. Association of NLRX1 with TBK1 and IKK pathways following LPS treatment

A. Raw 264.7 cells were treated with vehicle or 200ng/ml LPS overnight and Cell lysates were subjected to immunoprecipitation using mouse anti-NLRX1 antibody or mouse IgG.

The immunoprecipitates were separated by SDS-PAGE and blotted with p-TBK1, STING and NLRX1 antibodies. The blots represent best of the replicates (n=2).

B. Co-IP of endogenous NLRX1 with IKKα/β, IκB and TRAF6. Cell treatment, co-IP and Western blot (WB) were performed as described above. The blots represent best of the replicates (n=2).

Supplementary figure S7. Mitochondrial respiration following NLRX1 KD

PMVECs were transfected with 50 nM NLRX1 (red) or control siRNA (blue) for 72 hours. Mito Stress test was performed following NLRX1 KD.
